# Genetic association between CDKN2B-AS1 polymorphisms and the susceptibility of primary open-angle glaucoma (POAG): a meta-analysis from 21,775 subjects

**DOI:** 10.1007/s11845-021-02794-x

**Published:** 2021-10-14

**Authors:** Shanshan Liu, Siwen Chen, Tongtong Niu

**Affiliations:** Department of Ophthalmology, The Fourth People’s Hospital of Shenyang, Huanggu District, 20 Huanghe South Street, Shenyang, 11031 China

**Keywords:** CDKN2B-AS1, Meta-analysis, Polymorphism, Primary open-angle glaucoma

## Abstract

**Background:**

Primary open-angle glaucoma (POAG) is affected by both genetics and environmental factors. CDKN2B-AS1 polymorphisms have been reported to be involved in the pathogenesis of POAG. However, the results of the genetic associations between the CDKN2B-AS1 polymorphisms and POAG risk were inconclusive.

**Aims:**

This study aimed to evaluate the correlation of CDKN2B-AS1 polymorphisms and POAG susceptibility using a meta-analysis.

**Methods:**

Meta-analysis was performed by searching PubMed, Web of science, the Cochrane database of system reviews, CNKI, and Embase databases. The relationship of CDKN2B-AS1 rs4977756, rs10120688, rs2157719, and rs7049105 polymorphisms and POAG risk was evaluated by the odds ratios (ORs) and 95% confidence intervals (CIs).

**Results:**

Eleven studies with 8290 cases and 13,485 controls were included in the present meta-analysis. The alleles of rs4977756 and rs10120688 significantly increased the risk of POAG (rs4977756: OR = 1.20, 95%CI = 1.03–1.39, *p* = 0.02; rs10120688: OR = 1.36, 95%CI = 1.29–1.44, *p* < 0.00001). As for ethnicity, rs4977756 polymorphism significantly increased POAG risk in Caucasians (OR = 1.33, 95%CI = 1.12–1.57, *p* = 0.0009), but not in Asians. In addition, the rs2157719 allele was significantly associated with POAG risk in Asians (OR = 0.66, 95%CI = 0.55–0.80, *p* < 0.0001), but not in Caucasians (*p* > 0.05).

**Conclusions:**

The CDKN2B-AS1 rs4977756 might increase the POAG risk in Caucasian population, and rs2157719 might decrease the POAG risk in Asian population, while rs10120688 might increase the risk of POAG.

## Introduction

Glaucoma is one of the leading causes of blindness worldwide, especially in South Africa characterized by progressive damage of retinal ganglion cells, optic nerve head excavation, and visual field loss [1–5]. Primary open-angle glaucoma (POAG) is one of the most common type of glaucoma with a 4- to fivefold higher risk in South African than that in Caucasian populations [6, 7]. Although the POAG represents the most prevalent form of glaucoma, the pathogenesis and factors determining the disease progression are poorly understood. Risk factors including increased age, elevated intraocular pressure (IOP), African ancestry, and family history were identified in previous studies [8–10]. However, the underlying causes of these risk factors remain obscure. Additionally, the genetic factors have illustrated to play an important role in the development of POAG. Numbers of genome-wide association studies (GWAS) on the genetic association of POAG have identified multiple genomic loci in 7q31.1 (caveolin 1 (CAV1)/caveolin 2 (CAV2)) [11, 12], 1q24.1 (transmembrane and coiled-coil domains 1, TMCO1) [13], 14q23 (sin oculis homeobox 1/sin oculis homeobox 6, SIX1/SIX6) [14], and 9p21.3 (cyclin-dependent kinase inhibitor 2B antisense noncoding RNA, CDKN2B-AS1) [15] and genes including cytochrome P450 family 1 subfamily B polypeptide 1 (CYP1B1) [16], WD repeat domain 36 (WDR36) [17], TANK-binding kinase 1 (TBK1) [18], and galactosylceramidase (GALC) [19] in African, Caucasian, and Asian populations.

CDKN2B-AS1 is an antisense RNA that may influence the nearby CDKN2A and CDKN2B genes via regulatory mechanisms [20] and was determinate to be a genetic susceptibility locus for several age-related complex diseases including POAG [21, 22]. Recently, several GWA studies have identified a number of POAG-associated single-nucleotide polymorphisms (SNPs) such as rs4977756, rs10120688, rs2157719, and rs7049105 [23, 24]. Burdon et al. revealed CDKN2B-AS1 rs4977756 can significantly increase the risk of POAG in a cohort of 590 cases and 3956 controls in Australia [23]. Ng et al. also illustrated the genetic association between the CDKN2B-AS1 rs4977756 and POAG in Australians [25]. It is suggested that the stronger genetic signals at the 9p21 locus among females may contribute to the observed sex bias for POAG. However, several studies on the association between CDKN2B-AS1 rs4977756 and POAG in Chinese [26], Japanese [27], Pakistan [28], and American Caucasian [29] populations have shown inconsistent results. For limited studies and relatively small sample size in the correlation of CDKN2B-AS1 rs10120688, rs2157719, and rs7049105 and POAG, inconclusive results were observed.

Meta-analysis is powerful to obtain a more precise conclusion that was inconclusive in previous individual study. Considering the inconclusive results and limited sample size in previous studies, we performed a meta-analysis to further evaluate the genetic associations between CDKN2B-AS1 rs4977756, rs10120688, rs2157719, and rs7049105 polymorphisms and the susceptibility of POAG.

## Materials and methods

### Search strategy

The relevant literatures published as of April 1, 2021 were searched in PubMed, Chinese National Knowledge Infrastructure (CNKI), CQVIP, Chinese Biomedical Literature Database (CBM), Web of science, Embase, and Wanfang database. “cyclin-dependent kinase inhibitor 2B antisense RNA 1” or “CDKN2B-AS1” and “polymorphism” or “single nucleotide polymorphisms” or “SNPs” and “Primary Open-Angle Glaucoma” or “Open-Angle Glaucoma” or “POAG” or “OAG” were used as search terms. At the same time, the references included and related reviews were reviewed. Language is unlimited.

### Inclusion and exclusion criteria

The following criteria were used for the literature inclusion: (1) papers should concern CDKN2BAS polymorphism and glaucoma risk, (2) case–control and/or cohort designed studies, (3) contained SNP genotype data both in case and control groups, (4) adequate data for the calculation of odds ratios (ORs) and 95% confidence intervals (CIs), (5) the genotype distribution in control groups was in Hardy–Weinberg equilibrium (HWE). In addition, studies were excluded when they were (1) studies that contained overlapping data with other literatures; (2) data came from case-reports, reviews, or abstracts; (3) not case–control and/or cohort designed studies; (4) genotype frequencies were unavailable; and (5) the control group did not confirm to HWE.

### Data extraction

Liu SS and Chen SW independently extracted all data from each eligible study. Disputes was resolved by discussion. The following information from each study were extracted: the name of first author, publication, ethnicity, mean age, gender, type of glaucoma, mean intraocular pressure (IOP) (mm Hg), mean vertical cup-to-disc ratio (VCDR), and the number of cases and controls.

### Statistical analysis

Data was processed with RevMan 5 (Oxford, UK) and STATA12.0. The association between CDKN2B-AS1 polymorphisms and POAG risk was evaluated by pooled OR) and 95%CI. The significance of the pooled OR was assessed by the Z test. *I*^2^ was used to evaluate the heterogeneity between studies. If *I*^2^ was less than 50% (*p* > 0.05), the combined effect value OR and its 95%CI were calculated using the fixed-effect model; otherwise, the random effects model was used. Subgroup analyses were conducted based on ethnicity. Egger’s test and Begg’s test were used to evaluate and analyze the publication bias of each study. Meta-regression analysis was used to analyze the potential sources of inter-study heterogeneity. *p* value < 0.05 was considered to be significant difference.

## Results

### The characters of eligible studies

**Fig. 1:**
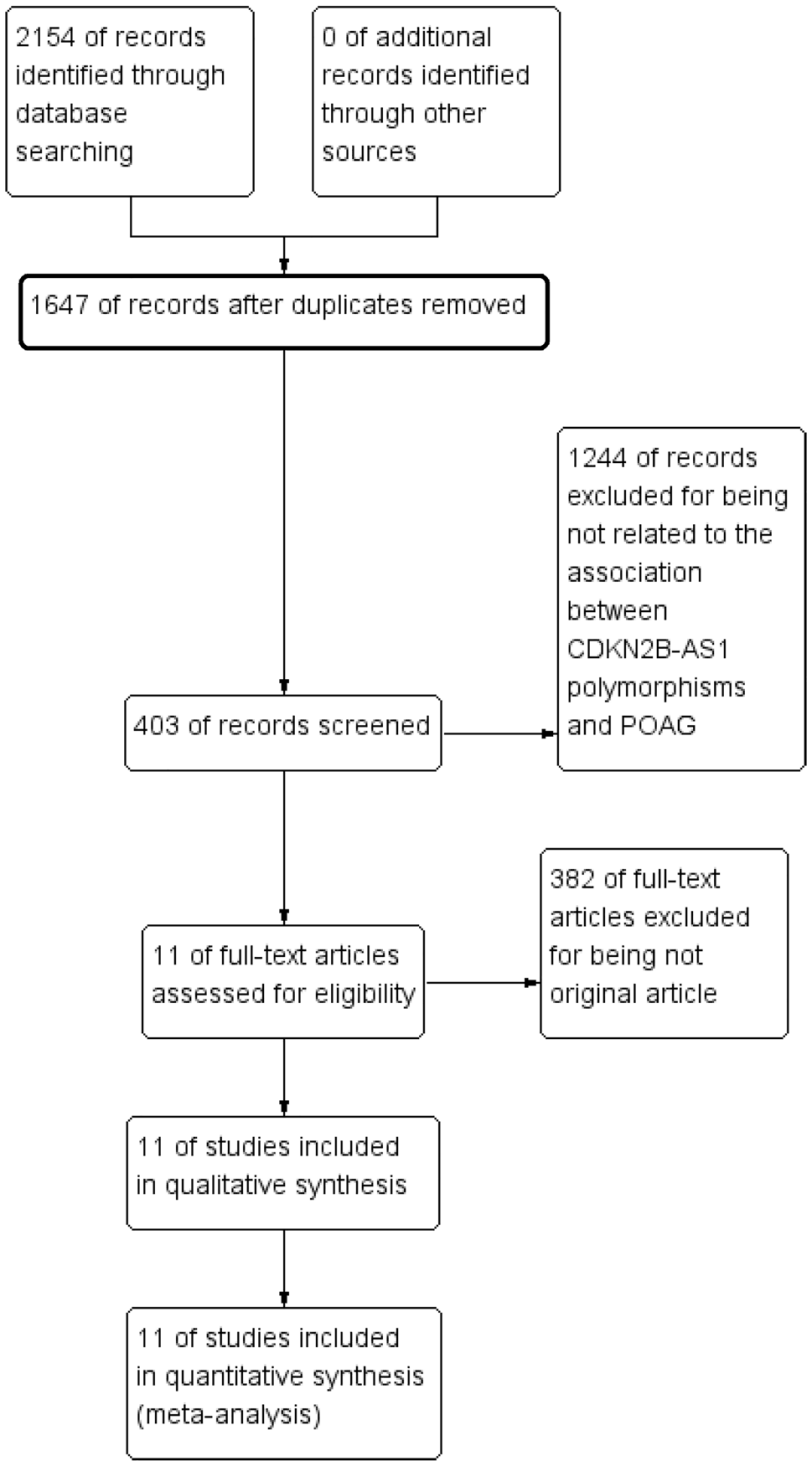
PRISMA flow chart of studies inclusion and exclusion

After an exhaustive search, a total of 2154 articles were retrieved from electric databases. According to the inclusion and exclusion criteria, we excluded 507 duplicated studies, 382 not original studies, and 1244 not case–control designed studies (Fig. 1). Finally, 11 studies with 8290 cases and 13,485 controls were enrolled in the present meta-analysis [15, 20, 23, 25–29, 39–41]. Among these articles, 7 studies refer to rs4977756, 3 studies refer to rs10120688 and rs2157719 respectively, and 2 studies refer to rs7049105. Furthermore, 4 studies were conducted on Asian populations, and 7 were on Caucasian populations. The basic information and genotype distribution of the included literatures are shown in Table 1.

**Table1:** The characters of included studies

First author	Year	Ethnicity	Age (year ± SD)	Gender (Male%)	Mean IOP (mm Hg)	Mean VCDR	Case	Control	Allelic model	
									Case	Control
Burdon	2014	Australian	68.9 ± 7.9/63.8 ± 8.3	27/43	17.6 ± 2.8/15.9 ± 2.6	0.53 ± 0.11/0.42 ± 0.12	67	1919	92	2268
Burdon-2	2011	Australian	72.0 ± 13.0/64.9 ± 12.4	48.9/52.6	NA	NA	892	4,582	1195	5498
Cao	2012	American	67.6 ± 11.8/62.1 ± 12.4	44.9/68.6	22.5 ± 7.0/16.3 ± 3.5	NA	272	165	181	118
Chen	2015	Chinese	48.81 ± 16.28/53.37 ± 14.83	67.76/40.26	30.10 ± 11.37/14.85 ± 2.84	0.85 ± 0.12/0.34 ± 0.11	1157	934	1819	1444
Kimura	2015	Japanese	56.2 ± 14.2/54.7 ± 13.9	46.9/33.0	NA	NA	247	276	378	409
Micheal	2014	Pakistan	54.6 ± 1.4/NA	52.0/NA	NA	NA	268	233	395	351
Ng	2016	Australian	60.6 ± 14.3/55.3 ± 8.7	47.3/43.5	27.12 ± 11.25/17.29 ± 3.27	0.88 ± 0.12/0.47 ± 0.13	2241	3176	3093	3786
Burdon-1	2011	Australian	62.4 ± 14.0/NA	46.8/NA	26.5 ± 10.0/NA	0.87 ± 0.13/NA	1201	595	1337	578
Nunes	2017	Brazil	NA	NA	NA	NA	310	247	119	126
Zanon-Moreno	2017	Spain	69.1 ± 9.0/67.7 ± 11.1	41.5/46.5	25.3 ± 3.6/16.8 ± 2.5	NA	391	383	372	310
Mori	2016	Japanese	62.6 ± 13.6/56.5 ± 14.0	47.8/36.7	NA	NA	1244	975	1689	1218

*SD* standard deviation, *NA* not available, *IOP* intraocular pressure, *VCDR* vertical cup-to-disc ratio

### Results of meta-analysis

**Fig. 2:**
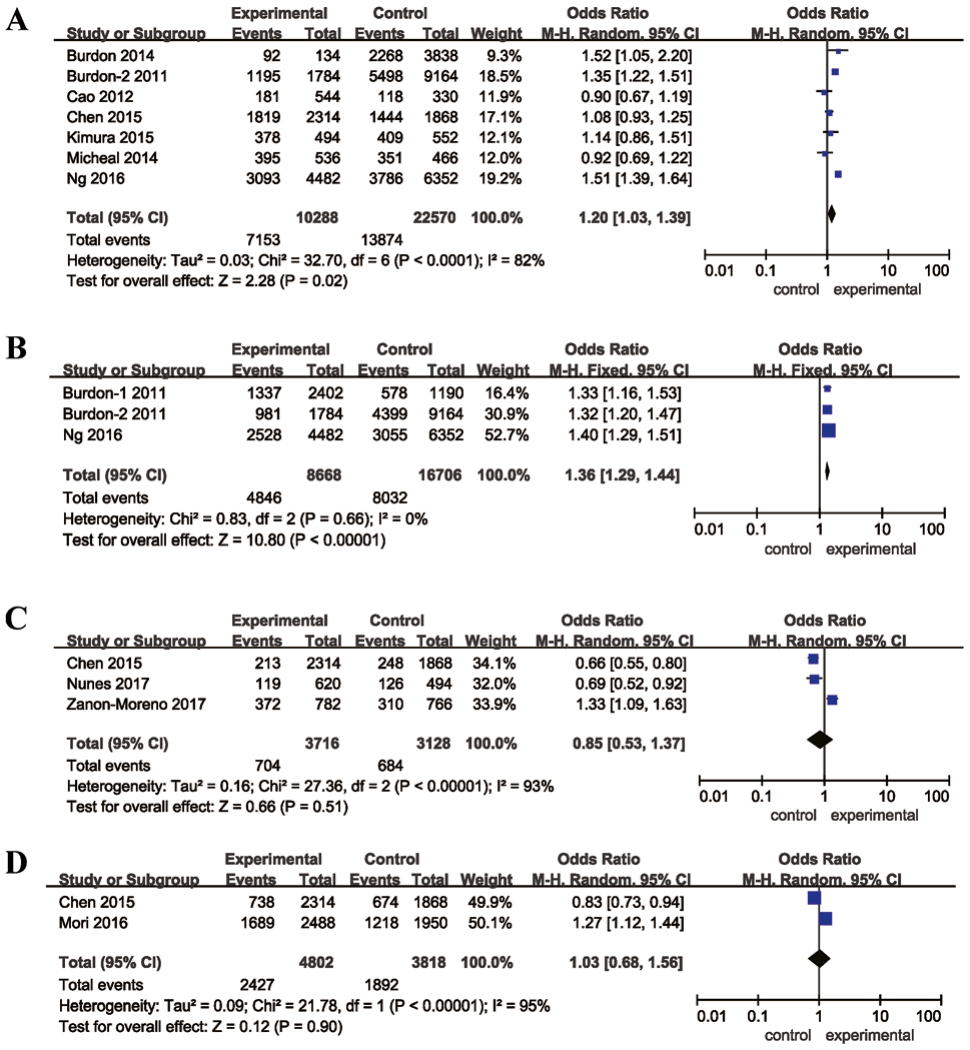
Forest plots of odds ratios for the association between the alleles of CDKN2B-AS1 polymorphisms and POAG. **A** rs4977756; **B** rs10120688; **C** rs2157719; **D** rs7049105

The results of the meta-analysis preformed in the current study are presented in Table 2. The allelic models of rs4977756 and rs10120688 significantly increased the risk of POAG (rs4977756: OR = 1.20, 95%CI = 1.03–1.39, *p* = 0.02; rs10120688: OR = 1.36, 95%CI = 1.29–1.44, *p* < 0.00001). Subgroup analysis based on ethnicity revealed that the allelic model of rs4977756 significantly increased the POAG risk in Caucasians (OR = 1.33, 95%CI = 1.12–1.57, *p* = 0.0009), but not in Asians (OR = 1.06, 95%CI = 0.94–1.19, *p* = 0.34). In addition, the allelic model of rs2157719 was associated with decreased risk of POAG in Asian population (OR = 0.66, 95%CI = 0.55–0.80, *p* < 0.0001), but not in Caucasian population (OR = 0.97, 95%CI = 0.51–1.84, *p* = 0.93). Subgroup analysis based on ethnicity was canceled for lack of sufficient data in rs10120688 and rs7049105 (Fig. 2). Moreover, the relationship of the dominant and recessive models of the CDKN2B-AS1 rs4977756, rs10120688, rs2157719, and rs7049105 polymorphisms and POAG susceptibility was canceled for lack of data.

**Table 2:** Combined results of the association between CDKN2B-AS1 polymorphisms and POAG risk

Polymorphisms	Minor allele	Subgroups	Number of studies	Test of association			Model	Test of heterogeneity	
				OR	95% CI	p-Value		*p* value	*I*^2^ (%)
rs4977756	A	Overall	7	1.20	[1.03,1.39]	0.02	R	< 0.0001	82
		Caucasian	4	1.33	[1.12, 1.57]	0.0009	R	0.005	77
		Asian	3	1.06	[0.94, 1.19]	0.34	F	0.53	0
rs10120688	A	Overall	3	1.36	[1.29, 1.44]	< 0.00001	F	0.66	0
rs2157719	G	Overall	3	0.85	[0.53, 1.37]	0.51	R	< 0.00001	93
		Caucasian	2	0.97	[0.51, 1.84]	0.93	R	0.002	93
		Asian	1	0.66	[0.55, 0.80]	< 0.0001	–	–	–
rs7049105	A	Overall	2	1.03	[0.68, 1.56]	0.90	R	< 0.00001	95

*CDKN2B-AS1* cyclin-dependent kinase inhibitor 2B antisense noncoding RNA, *OR* odd ratio, *CI* confidence interval, *R* random model; *F* fixed model, – not available

### Heterogeneity

Heterogeneity was found for the rs4977756 (*I*^2^% = 82, *p* < 0.0001), rs2157719 (*I*^2^% = 93, *p* < 0.00001), and rs7049105 (*I*^2^% = 95, *p* < 0.00001) in overall analysis. Significant heterogeneity was detected in subgroup analysis based on ethnicity (Caucasians: rs4977756: *I*_2_% = 77, *p* = 0.005; rs2157719: *I*_2_% = 93, *p* = 0.002). The significant heterogeneity in rs4977756 was primarily presented by Burdon et al. and Ng et al. No significant heterogeneity was detected after removal these studies (*I*_2_ = 37%, *p* > 0.05). Moreover, the significant heterogeneity in rs2157719 was primarily presented by Zanon-Moreno et al. And no significant heterogeneity was detected after removal these studies (*I*_2_ = 0%, *p* > 0.05) (Table 2).

### Sensitive analysis and publish bias

**Fig. 3:**
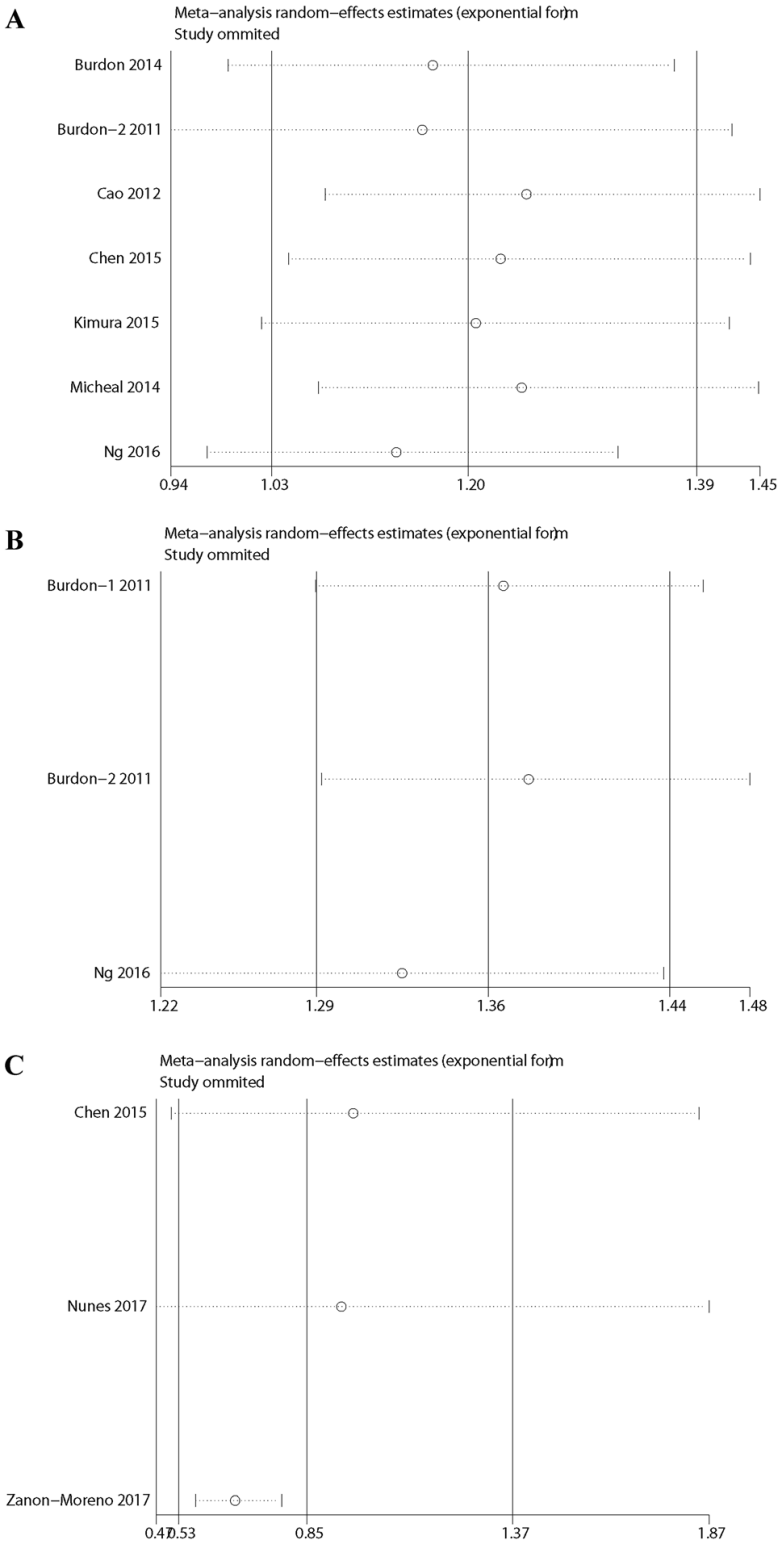
Sensitivity analyses between allelic models of CDKN2B-AS1 polymorphisms and POAG. **A** rs4977756; **B** rs10120688; **C** rs2157719

**Fig. 4:**
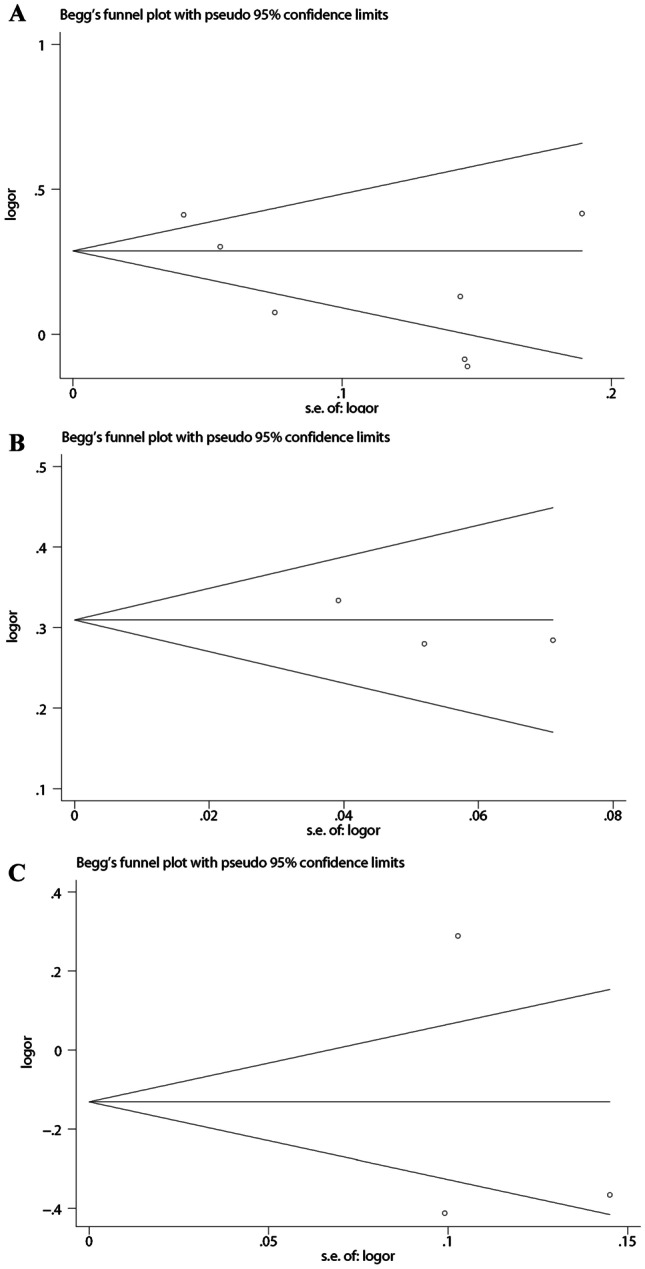
Publication bias of literatures for CDKN2B-AS1 polymorphisms were tested by Begg’s funnel plot. **A** rs4977756; **B** rs10120688; **C** rs2157719

There was no deviation from Hardy–Weinberg equilibrium or OR 3.0 in the 11 studies on CDKN2B-AS1 rs4977756, rs10120688, rs2157719, and rs7049105 polymorphisms in the present meta-analysis. Thus, the association between CDKN2B-AS1 rs4977756, rs10120688, rs2157719, and rs7049105 and POAG was stable and reliable. As shown in Table 3 and Figs. 3 and 4, no evidence of publication bias was found by using Begg’ s test and Egger’ s test.

**Table 3:** Begg’s linear regression test for funnel plot asymmetries of CDKN2B-AS1 positions

SNPs	rs4977756	rs10120688	rs2157719	rs7049105
*p* value	0.082	0.399	0.834	NA
95%CI	− 6.606928–0.5544333	− 18.67542–15.0103	− 238.4966–228.7118	NA

*CDKN2B-AS1* cyclin-dependent kinase inhibitor 2B antisense noncoding RNA, *CI* confidence interval, *NA* not available

## Discussion

In the present study, we have assessed the genetic association of CDKN2B-AS1 rs4977756, rs10120688, rs2157719, and rs7049105 and the risk of POAG and found that the CDKN2B-AS1 rs4977756 and rs10120688 can significantly increase the risk of POAG. However, the CDKN2B-AS1 rs4977756 was associated with POAG risk only in Caucasian, but not in Asian. In addition, the rs2157719 decreases the POAG risk only in Asian. No association between rs7049105 and the risk of POAG was detected.

GWAS data have identified significant associations between several genes in chromosome 9p21 and multiple common diseases [30–32]. CDKN2B-AS1 is a non-coding gene (also known as ANRIL) with an unknown function, located on chromosome 9p21.3 [33]. Multiple tissue-specific splice variants of this gene have been reported. This gene is likely to play a role in regulating expression of genes through epigenetic mechanisms. CDKN2B-AS1 polymorphisms were illustrated to be associated with coronary artery disease (CAD) [34] and type 2 diabetes mellitus risk initially [35]. Subsequently, the polymorphisms in CDKN2B-AS1 were identified to be significantly associated with the development of POAG [29]. However, little is known about the biological meanings underlying this locus. Song et al. have shown that 33 enhancers were identified in 9p21, some of which being within CDKN2B-AS1 [36]. Thus, it is suggested that the variants in this gene would probably affect the expression level of the downstream genes CDKN2A and CDKN2B, which is response to the elevated IOP in glaucoma. Altered gene expression may also affect cell cycle regulation and lead to a tendency toward apoptosis of retinal ganglion cells. The cells of POAG patients carrying the risk alleles of CDKN2B-AS1 gene may be more sensitive to IOP. Moreover, the variants in this gene might also affect the distant genes that correlated with the complex pathogenesis of glaucoma.

The rs4977756 is mapped 59 kb telomeric to CDKN2B within a 122-kb region of LD at 9p21.3, which encompasses the CDKN2A-CDKN2B tumor suppressor genes and was shown to be associated with the risk of glioma [37, 38]. In this meta-analysis, a total of 5144 cases and 11,285 controls from 7 publications were included. And the CDKN2B-AS1 rs4977756 polymorphism was significantly associated with increased POAG risk. To our knowledge, this is the first time a significant genetic association between CDKN2B-AS1 rs4977756 polymorphism and the susceptibility of POAG was detected using a meta-analysis. As for ethnicity, rs4977756 polymorphism was associated with increased risk of POAG in Caucasians, but not in Asians. Interestingly, GWAS data has demonstrated that the rs4977756 polymorphism was a risk factor for POAG in Japanese, which may suggest that the CDKN2B-AS1 locus in previous study using a Japanese population seemed to be shared with the Caucasian subjects, but not with the Chinese and other Asian populations. Thus, the CDKN2B-AS1 rs4977756 for POAG is still ethnicity related.

The current meta-analysis had various limitations. Firstly, the ethnicity included in the present meta-analysis was relatively limited. Subgroup analysis was only divided into Caucasian and Asian. Considering the significant influence of African ancestry in the pathogenesis of POAG, studies on the association between CDKN2B-AS1 polymorphisms and POAG risk in other ethnicities such as African and Latin American are necessary in the future. Secondly, age and gender were shown to be involved in the pathogenesis of POAG. However, we could not perform further stratification analyses based on age and gender due to limited data. Thirdly, we failed to assess the potential influence of genetic and environmental factors, as well as the gene–gene and gene-environment interactions on POAG for lack of relevant data. Fourthly, CDKN2B-AS1 rs4977756, rs10120688, rs2157719, and rs7049105 polymorphisms would not be enough to explain the associations between CDKN2B-AS1 gene and POAG risk. Fifthly, the dominant and recessive models of these polymorphisms were not available for lack of relevant data.

## Conclusions

The CDKN2B-AS1 rs4977756 might increase the POAG risk in Caucasian population. rs10120688 might be associated with an increased risk of POAG. rs2157719 might decrease the risk of POAG in Asian population. To confirm these results, a larger number of subjects with different ethnicity are necessary to explore the role of CDKN2B-AS1 rs4977756 and rs10120688 in POAG.

## Data Availability

The data used to support the founding of this study are included within the article.
